# The Effects of l-Arginine, Alone and Combined with Vitamin C, on Mineral Status in Relation to its Antidiabetic, Anti-Inflammatory, and Antioxidant Properties in Male Rats on a High-Fat Diet

**DOI:** 10.1007/s12011-013-9867-5

**Published:** 2013-12-01

**Authors:** Joanna Suliburska, Paweł Bogdanski, Zbigniew Krejpcio, Danuta Pupek-Musialik, Anna Jablecka

**Affiliations:** 1 Department of Human Nutrition and Hygiene, Poznan University of Life Sciences, Wojska Polskiego 31, 60-624 Poznan, Poland; 2 Department of Internal Medicine, Metabolic Disorders and Hypertension, Poznan University of Medical Sciences, Poznan, Poland; 3 Department of Clinical Pharmacology, Poznan University of Medical Sciences, Poznan, Poland

**Keywords:** l-arginine, Vitamin C, High-fat diet, Minerals, Insulin, Antioxidant status, Inflammation

## Abstract

The aim of this study was to evaluate the influence of the intake of l-arginine alone and of l-arginine with vitamin C on mineral concentration in rats fed with a high-fat diet, and to assess the lipid glucose, insulin, and total antioxidant status (TAS) and tumor necrosis factor (TNF) alpha serum levels that result. Wistar rats were assigned to groups fed with either a standard control diet (C), a diet high in fat (FD), a diet high in fat with l-arginine, or a diet high in fat with l-arginine and vitamin C. After 6 weeks, the length and weight of the rats were measured, and the animals were euthanized. The liver, spleen, kidneys, pancreas, heart, and gonads were collected, as were blood samples. The total serum cholesterol, triglyceride, fasting glucose, insulin, TAS, and TNF alpha levels were measured. The tissue calcium, magnesium, iron, zinc, and copper concentrations were determined. It was found that l-arginine supplementation diminished the effect of the modified diet on the concentration of iron in the liver and spleen and of copper in heart. At the same time, it was observed that l-arginine supplementation reduced the effect of the high-fat diet on insulin, TNF alpha, and TAS. The combination of l-arginine and vitamin C produced a similar effect on the mineral levels in the tissues as did l-arginine used alone. Moreover, positive correlations between serum insulin and iron in the liver, between TNF alpha and iron in the liver, and between TNF alpha and copper in the heart were observed. The level of TAS in serum was inversely correlated with the copper level in the heart and the iron level in the liver. We concluded that the beneficial influence of l-arginine on insulin, TAS, and TNF alpha serum level is associated with changes in the iron and copper status in rats fed with a high-fat diet. No synergistic effect of l-arginine and vitamin C in the biochemical parameters or in the mineral status in rats fed with the modified diet was observed.

## Introduction

Unbalanced diets have contributed to the increased prevalence of obesity and other metabolic disorders in the modern world [1]. Experimental and clinical research have shown that high-fat diets lead to increased serum glucose, insulin, and triglyceride levels and promote insulin resistance, oxidative stress, and inflammation [2, 3]. Moreover, it has been observed that both the western diet (high in fat, sodium, and sugar) and metabolic disorders are associated with impairments in mineral status [4]. An increasing amount of evidence suggests that mineral disturbances are involved in complex pathogenesis of obesity-related complications. An association has been demonstrated between mineral status on the one hand and antioxidant markers, inflammation markers, glucose metabolism, and lipid metabolism on the other. Recent studies have indicated that it is worth considering mineral control and supplementation in some populations, including patients with obesity and related diseases [5–7].

Due to the high incidence of metabolic disorders, compounds that might prevent or treat them are sought for. It is known that a significant deficiency in nitric oxide (NO) is one of the disorders contributing to metabolic syndrome. The substrate for NO synthesis is l-arginine. Increasingly, the evidence points to the potential benefits of the use of l-arginine for decreasing oxidative stress, decreasing blood pressure, reducing the severity of atherosclerosis, and improving insulin sensitivity [8–11]. Its precise mechanism, however, has not yet been fully determined. The potential use of l-arginine in patients with obesity and other metabolic disorders seems to be promising, especially when combined with other substances with similar properties—for example, antioxidants [12–14]. There is a lack of experimental research assessing l-arginine supplementation under the condition of metabolic disorders caused by a diet high in fat in rats, and also the influence of l-arginine on mineral status in rats.

Considering the beneficial properties of l-arginine, we have carried out a study to evaluate the influence of l-arginine alone and with vitamin C on mineral status in rats, and to assess the resulting changes in lipid, glucose, insulin, antioxidant, and inflammation status in rats fed with a diet that promotes metabolic disorders.

## Materials and Methods

All animal procedures were performed according to approved protocols and in accordance with the recommendations for the proper care and use of laboratory animals. The protocol of the study was approved by the local bioethical commission in Poznań (approval no. 20/2011).

### Animals

The experiment was performed using male Wistar rats (8 weeks old, weighing 191 ± 10 g). The rats were purchased from the Department of Toxicology at the Medical University of Poznan, Poland. All rats were housed individually in polycarbonate cages in a room controlled for temperature and humidity on a 12-h light, 12-h dark cycle, designed for the purpose of the study. The indoor animal house temperature was 21 ± 2 °C and the relative humidity was 60 ± 5 %. The animals were housed under standard conditions.

### Experimental Design

The experiment was performed using 24 male Wistar rats. After a 5-day period of adaptation to laboratory conditions, the rats were randomly divided into four equally sized groups:

- Group 1, the control (C, *n* = 6): untreated rats, allowed free access to standard diet
- Group 2, high-fat diet (FD, *n* = 6): received a high-fat diet
- Group 3, arginine group (FD + ARG; *n* = 6): received a high-fat diet containing 20 g/kg diet of l-arginine
- Group 4, arginine and vitamin C group (FD+ARG+VITC; *n* = 6): received a high-fat diet containing 20 g/kg diet of l-arginine and 100 mg/kg diet of vitamin C

The animals were fed with either a standard semisynthetic diet based on the AIN-93 M diet [15] or a high-fat diet modified with amounts of fat and sodium chloride. The full composition of the diets is presented in Tables 1 and 2. l-arginine (Curtis Healthcare, Warsaw, Poland) was administered in the diet at a dose of 20 g/kg diet for 6 weeks. Vitamin C (Biofarm, Poznan, Poland) was administered in the diet at a dose of 100 mg/kg diet for 6 weeks. The appropriate value of wheat starch was reduced in the diet—20 g for l-arginine and 100 mg for vitamin C.

**Table 1:** Ingredient and nutrient composition of the diets (grams per kilogram diet)

Ingredient	Standard diet	High fat diet
Casein	140	140
Wheat starch	625	430
Sucrose	100	100
Potato starch	50	50
Vitamin mixture^a^	10	10
Mineral mixture^b^	35	35
Sunflower oil	40	40
Lard	-	160
Sodium chloride	-	35
Total energy (kcal/100 g diet)	420	515
Total protein (% of energy)	18	18
Total fat (% of energy)	9	39

^a^Composition of vitamin mixture (g/kg mix): nicotinic acid (3), Ca pantothenate (1.6), pyridoxine (0.7), thiamin (0.6), riboflavin (0.6), folic acid (0.2), biotin (0.02), vitamin B_12_ (0.003), vitamin E (500 IU/g), vitamin A (500,000 IU/g), vitamin D_3_ (400,000 IU/g), vitamin K_1_ (0.08), choline bitartrate (200), powdered sucrose (777.15)

^b^Composition of mineral mixture (g/kg mix): calcium carbonate (357), potassium phosphate monobasic (250), potassium citrate (28), sodium chloride (74), potassium sulfate (46.6), magnesium oxide (24), ferric citrate (6.06), zinc carbonate (1.65), sodium meta-silicate⋅9H_2_0 (1.45), manganous carbonate (0.64), cupric carbonate (0.30), chromium chloride⋅6H_2_0 (0.147), boric acid (0.0816), sodium fluoride (0.0635), nickel chloride⋅6H_2_0 (0.0578), lithium sulfate⋅H_2_0 (0.0263), sodium selenate anhydrous (0.0103), potassium iodate (0.010), ammonium paramolybdate⋅4H_2_0 (0.0795), ammonium vanadate (0.066), powdered sucrose (209.758)

**Table 2:** Minerals concentration in the diets

Parameter	Group			
	C	FD	FD + ARG	FD + ARG + VITC
Ca (g/kg)	11.78 ± 0.07	11.60 ± 0.28	11.72 ± 0.40	11.82 ± 0.51
Mg (mg/kg)	538.27 ± 6.48	543.03 ± 15.03	535.54 ± 11.80	545.27 ± 14.12
Fe (mg/kg)	43.20 ± 1.76	43.73 ± 3.84	44.18 ± 1.70	45.92 ± 1.52
Zn (mg/kg)	29.83 ± 4.90	31.65 ± 3.07	28.25 ± 1.70	30.89 ± 2.49
Cu (mg/kg)	5.72 ± 0.25	5.47 ± 0.34	5.52 ± 3.34	5.76 ± 0.29

*C* control group, *FD* high-fat diet group, *FD* + *ARG* high-fat diet with l-arginine group, *FD* + *ARG* + *VITC* high-fat diet with l-arginine and vitamin C group

All rats were provided ad libitum diet and distilled water for 42 days. Dietary intake was recorded daily, while body weight gain was monitored weekly before food distribution (Table 3).

**Table 3:** Final body mass, obesity index, and relative weight of tissues

Parameter	Group			
	C	FD	FD + ARG	FD + ARG + VITC
Diet intake (g/day)	21.5 ± 1.8	20.5 ± 1.2	20.8 ± 1.5	20.8 ± 1.7
Final body mass (g)	343.0 ± 34.7	345.8 ± 28.8	333.0 ± 36.3	323.2 ± 41.2
Obesity index	303.00 ± 34.68	292.65 ± 28.86	281.00 ± 36.28	303.17 ± 41.21
Liver (%of body mass)	3.06 ± 0.19	2.85 ± 0.17	2.80 ± 0.16	2.88 ± 0.17
Kidney (% of body mass)	0.71 ± 0.15	0.74 ± 0.03	0.72 ± 0.05	0.70 ± 0.03
Heart (%of body mass)	0.29 ± 0.01	0.32 ± 0.02	0.31 ± 0.02	0.30 ± 0.02
Spleen (% of body mass)	0.19 ± 0.02	0.16 ± 0.02	0.17 ± 0.02	0.17 ± 0.02
Pancreas(% of body mass)	0.34 ± 0.04	0.32 ± 0.01	0.30 ± 0.03	0.31 ± 0.02
Gonads (% of body mass)	0.94 ± 0.14	1.00 ± 0.12	1.04 ± 0.11	1.06 ± 0.14

*C* control group, *FD* high-fat diet group, *FD* + *ARG* high-fat diet with l-arginine group, *FD* + *ARG* + *VITC* high-fat diet with l-arginine, and vitamin C group; obesity index was calculated by dividing the cubic root of the body weight (grams) by the nasoanal length (millimeters) × 10^4^; the means are not significantly different (*P* > 0.05)

Over the whole course of the experiment, the animals were under veterinary supervision.

### Tissue and Serum Collection

At the end of the experimental period and following 16 h of fasting, the animals were weighed and anesthetized with a sodium thiopental injection (40 mg/kg body weight), and killed by cardiac puncture. The blood samples were collected in serum-separated tubes for biochemical studies. The blood was left to clot at room temperature for 30 min, and then centrifuged for 15 min at 2,500 rpm at 4 °C. The supernatant fluid was then separated. Serum samples were stored at −70 °C until analyzed. The liver, spleen, heart, kidneys, pancreas, and gonads were dissected, weighed, and kept frozen at −70 °C until mineral content analysis was performed. In all animals, the weight gain and the absolute and relative weight of the tissues was determined. The relative weight of tissues was defined as their percentage of body weight.

### Biochemical Measurements

Total cholesterol, triglyceride, and fasting glucose levels in serum were measured using commercial kits (Randox Laboratories Ltd., UK) in the diagnostic laboratory at Poznan, Poland. The concentration of cholesterol and triglycerides in serum was assayed by the enzymatic method (Siemens Healthcare Diagnostics, Erlangen, Germany). Serum glucose concentration was assayed by an enzymatic method involving hexokinase and glucose-6-phosphate dehydrogenase (Siemens Healthcare Diagnostics, Erlangen, Germany).

The plasma concentration of insulin was determined by an enzyme-linked immunosorbent assay, strictly according to the manufacturer’s instructions (Demeditec Diagnostic GmbH, Kiel–Wellsee, Germany).

Insulin resistance was estimated using homeostasis model assessment according to the following formula: insulin resistance index = fasting insulin (in micrograms per liter) × fasting glucose (in milligrams per deciliter)/405.

The plasma level of tumor necrosis factor (TNF) alpha was determined by enzyme-linked immunosorbent assay, strictly according to the manufacturer’s instructions (R&D Systems, Minneapolis, USA).

The total antioxidant status (TAS) was measured using a TAS Randox kit (Randox Laboratories Ltd., UK) and spectrophotometry (SPECORD M40, Carl Zeiss, Jena, Germany).

### Determination of Minerals

The calcium, magnesium, iron, zinc, and copper contents of the tissues were determined after digesting the tissues in 65 % (w/w) spectra pure HNO_3_ (Merck) in the Microwave Digestion System (MARS 5, CEM Corp., USA). The concentrations of the minerals in the mineral solutions were then measured using flame atomic absorption spectrometry (AAS-3, Carl Zeiss, Jena, Germany), at the following wavelengths: 422.7 nm for calcium, 285.2 nm for magnesium, 248.3 nm for iron, 213.9 nm for zinc, and 324.8 nm for copper. The accuracy of the method was verified with certified reference materials (pig kidney BCR no. 186, Brussels) and was 92 % for calcium, 98 % for magnesium, 92 % for iron, 95 % for zinc, and 102 % for copper. The concentrations of minerals in the tissues were expressed as wet weights.

### Statistical Analysis

A detailed statistical analysis was performed using Statistica for Windows 10.0. The results were expressed as arithmetic means and standard errors. Continuous variables were assessed for normal distribution with the use of the Shapiro–Wilk test. Differences between means were analyzed by one-way analysis of variance, followed by a post hoc Tukey’s test. A value of *P* < 0.05 was regarded as representing a significant difference.

## Results

The average intakes of diet and obesity indices were found to be comparable in all groups (Table 3). The relative masses of the tissues (as percentages of body mass) were also comparable among the groups (Table 3).

The results presented in Table 4 demonstrate that 6 weeks of high-fat diet resulted in a significant increase in serum insulin and TNF alpha concentration and a decrease in TAS serum levels, while l-arginine supplementation (both supplemented alone and in combination with vitamin C) diminished the effects of the high-fat diet on insulin, TNF alpha, and TAS. It was observed that l-arginine with vitamin C had a slightly better influence on the biochemical parameters than did l-arginine alone. Serum glucose, cholesterol, and triglyceride levels were comparable among the groups.

**Table 4:** Biochemical parameters in rats

Parameter	Group			
	C	FD	FD + ARG	FD + ARG + VITC
Glucose (mmol/l)	5.10 ± 0.66	5.22 ± 0.76	4.82 ± 0.60	4.91 ± 0.56
T-CHOL (mmol/l)	2.00 ± 0.09	2.06 ± 0.15	1.90 ± 0.19	1.78 ± 0.13
TG (mmol/l)	0.38 ± 0.09	0.39 ± 0.10	0.40 ± 0.08	0.35 ± 0.08
Insulin (pmol/l)	54.10 ± 10.40^a^	113.43 ± 12.22^c^	85.51 ± 5.21^b^	62.35 ± 10.47^a,b^
TNF alpha (ng/ml)	5.27 ± 1.23^a^	8.13 ± 2.18^b^	7.33 ± 1.75^a,b^	6.18 ± 1.93^a,b^
TAS (mmol/l)	1.28 ± 0.12^b^	0.97 ± 0.21^a^	1.16 ± 0.10^a,b^	1.30 ± 0.08^b^

*C* control group, *FD* high-fat diet group, *FD* + *ARG* high-fat diet with l-arginine group, *FD* + *ARG* + *VITC* high-fat diet with l-arginine and vitamin C group, *T*-*ChOL* total cholesterol, *TG* triglycerides, *TAS* total antioxidant status, *TNF alpha* tumor necrosis factor alpha; means in the same line with different superscripts are significantly different (*P* < 0.05)

In Table 5, the results of the mineral concentration analyses of the tissues are given. It was found that the high-fat diet led to a significant increase in the iron concentration in the liver and in the copper level in the heart. At the same time, the level of iron in the spleen markedly decreased under the influence of the high-fat diet. l-arginine supplementation diminished the effect of the modified diet on the concentration of iron in the liver and spleen and copper in the heart (see Table 5).

**Table 5:** Mineral concentrations in tissues (μg/g wet wt.)

Parameter	Group			
	C	FD	FD + ARG	FD + ARG + VITC
Liver				
Ca	24.85 ± 2.60	24.27 ± 4.63	24.53 ± 3.23	32.40 ± 8.82
Mg	262.61 ± 14.93	270.73 ± 10.65	274.21 ± 7.33	246.95 ± 16.28
Fe	63.84 ± 15.05^a^	116.21 ± 18.51^b^	99.81 ± 20.62^a,b^	98.47 ± 20.69^a,b^
Zn	29.59 ± 1.50	30.70 ± 1.04	30.89 ± 1.20	30.39 ± 2.01
Cu	4.68 ± 0.32	4.93 ± 0.36	4.67 ± 0.27	4.58 ± 0.08
Spleen				
Ca	39.50 ± 4.59	34.02 ± 4.63	39.03 ± 4.83	38.45 ± 8.31
Mg	258.51 ± 18.58	258.19 ± 19.53	240.93 ± 6.12	245.54 ± 24.74
Fe	382.47 ± 65.09^b^	226.14 ± 38.66^a^	332.02 ± 41.75^a,b^	328.52 ± 46.25^a,b^
Zn	19.09 ± 1.68	19.84 ± 1.25	19.73 ± 0.63	19.77 ± 1.63
Cu	0.86 ± 0.07	0.81 ± 0.08	0.69 ± 0.08	0.76 ± 0.10
Heart				
Ca	27.55 ± 2.05	28.15 ± 3.85	27.63 ± 1.06	27.43 ± 2.92
Mg	237.32 ± 25.88	242.46 ± 25.05	242.99 ± 14.63	242.22 ± 14.11
Fe	78.15 ± 6.63	67.01 ± 1.38	71.74 ± 0.77	73.34 ± 4.15
Zn	14.76 ± 0.58	15.37 ± 0.37	15.34 ± 0.54	15.34 ± 0.66
Cu	5.00 ± 0.28^a^	8.37 ± 0.56^b^	5.15 ± 0.25^a^	4.91 ± 0.16^a^
Kidney				
Ca	31.48 ± 2.46	30.87 ± 2.62	32.26 ± 1.20	32.17 ± 1.46
Mg	224.89 ± 11.11	223.98 ± 8.74	225.77 ± 13.22	233.74 ± 11.91
Fe	39.53 ± 4.04	49.70 ± 7.96	42.12 ± 6.81	32.61 ± 2.18
Zn	20.96 ± 1.21	21.55 ± 0.65	20.56 ± 0.96	20.91 ± 1.23
Cu	6.76 ± 0.70	6.81 ± 1.17	6.09 ± 1.03	5.84 ± 0.40
Pancreas				
Ca	53.24 ± 5.09	68.74 ± 5.40	67.11 ± 2.61	65.80 ± 4.80
Mg	274.42 ± 17.17	272.80 ± 26.91	298.73 ± 14.53	278.74 ± 10.99
Fe	19.64 ± 0.87	18.29 ± 1.09	18.82 ± 2.39	20.30 ± 3.31
Zn	22.27 ± 2.48	20.53 ± 0.58	20.47 ± 1.36	20.40 ± 1.48
Cu	1.03 ± 0.13	0.91 ± 0.08	0.81 ± 0.11	0.78 ± 0.12
Gonads				
Ca	16.84 ± 3.22	15.58 ± 5.95	16.55 ± 2.83	16.77 ± 1.30
Mg	151.84 ± 20.82	154.62 ± 13.32	155.43 ± 31.28	151.71 ± 23.62
Fe	24.61 ± 3.48	24.14 ± 3.83	22.32 ± 2.23	22.01 ± 1.63
Zn	38.66 ± 4.79	34.77 ± 3.85	33.22 ± 1.64	31.04 ± 3.47
Cu	2.28 ± 0.43	1.82 ± 0.34	2.03 ± 0.60	1.68 ± 0.20

*C* control group, *FD* high-fat diet group, *FD* + *ARG* high-fat diet with l-arginine group, *FD* + *ARG* + *VITC* high-fat diet with l-arginine and vitamin C group; means in the same line with different superscripts are significantly different (*P* < 0.05)

The combination of l-arginine with vitamin C had a similar effect on the mineral level in the tissues as did l-arginine used alone.

Several correlations were found between serum insulin, TAS, and TNF alpha and iron and copper levels in all rats (Table 6). Serum insulin positively correlated with the iron level in the liver. The value of the TAS in serum was inversely correlated with the copper level in the heart and the iron level in the liver. Moreover, a positive correlation of TNF alpha level with the iron concentration in the liver, and also with the copper concentration in the heart, was observed.

**Table 6:** Significant correlation indexes between minerals and biochemical parameters (*P* < 0.05)

Parameters	r
Fe in liver—serum insulin	0.83
Cu in heart—TAS	−0.76
Fe in liver—TAS	−0.63
Fe in liver—TNF alpha	0.76
Cu in heart—TNF alpha	0.71

*r* Pearson correlation coefficient, *TAS* total antioxidant status, *TNF alpha* tumor necrosis factor alpha

## Discussion

In this study, we have demonstrated that 6 weeks of treatment with l-arginine diminishes the effect of the high-fat diet on insulin serum level, inflammation, and antioxidant status, and was associated with changes in the iron and copper status of rats. This is a new finding of the study. The influence of l-arginine supplementation in improving insulin status has also been observed in other experimental and clinical studies [9, 16]. In particular, it has been shown that l-arginine stimulates insulin secretion [17, 18]. In our previous study, we found that supplementation with l-arginine increases antioxidant status in obese patients [13]. The antioxidant effects of l-arginine could be due to the decreasing intensity of radical reactions or of antioxidative–enzyme induction in the body of rats on a high-fat diet. It is also known that l-arginine may reduce superoxide anion release from endothelial cells, and thus affect the lowering of oxidative stress [19, 20]. l-arginine may lower the amount of free oxygen radicals, and can thus inhibit tissue damage and decrease inflammation status in rats, which can promote negative metabolic changes. Increasing amounts of evidence confirm the anti-inflammatory and antioxidant properties of l-arginine [21–23].

In this study, it was also found that vitamin C did not significantly impact the effect of l-arginine on insulin, TAS, TNF alpha, or minerals in rats fed with the high-fat diet. The combination showed only slightly better effects on insulin, TNF alpha, and TAS. The results we obtained were not expected, especially as other studies have shown a beneficial synergistic effect of the administration of antioxidants with l-arginine [12, 22, 24, 25]. However, the conditions in these other studies differed from those obtained in our research. It is possible that a longer experimental period would reveal significant differences between the use of l-arginine alone and of l-arginine combined with vitamin C. Another explanation is possibly the lack of synergistic effect of vitamin C and arginine. Fan X. et al. [26, 27] have obtained strong evidence for that hypothesis that the accumulation of ascorbic acid oxidation products that show high reactivity with proteins is responsible for the formation of crystalline adducts and crosslinks in vivo. On one hand, various studies clearly suggest that vitamin C is beneficial for decreasing the risk of cataractogenesis, in particular under conditions that favor oxidant stress [28]. The proposed mechanisms include the scavenging of free radicals by the vitamin itself or in conjunction with glutathione. On the other hand, it has been observed that, under some clinical conditions, when the cell’s defenses are weakened by diabetes, end-stage renal disease, or poor nutrition, vitamin C can inflict damage [26]. It seems probable that the high-fat diet used in our experiment could predispose to carbonyl and oxidant stress, and is also associated with ascorbic acid oxidation products.

The results of this study show that the influence of l-arginine in rats, whether alone or with vitamin C, on TAS and TNF alpha is strongly associated with changes in iron and copper status. It was observed that the modified diet high in fat and sodium, which promoted negative metabolic changes, induced the displacement of iron ions from the spleen to the liver and the accumulation of copper ions in the heart. These changes in iron and copper levels in the liver and heart may negatively influence oxidative stress and inflammation in these tissues. Iron ions participate in the generation of free radicals, which may damage hepatic cells and decrease antioxidant status, while increasing inflammation in the body [29–31]. Similarly, the increase in copper concentration in the heart of the rats fed with the high-fat diet might induce oxidative stress in this tissue, and thus determine an inflammatory response [32].

In this study, it was found that l-arginine decreased the displacement of iron ions between the spleen and the liver and inhibited the accumulation of copper in the heart, in rats fed with the modified diet. The mechanism of the influence of l-arginine on iron and copper ions is so far unknown to us, though it may be connected with the antioxidative and anti-inflammatory properties of l-arginine [12]. It could also be linked to the fact that arginine is a substrate in the synthesis of nitric oxide. Other authors have suggested that NO may play an important role in iron metabolism [33]. Xiao et al. [33] found a negative correlation between plasma NO and iron concentration under conditions of l-arginine supplementation in exercised rats, such that increases in NO levels during exercise during l-arginine supplementation were found to lead to decreased catalytic iron concentration in the liver and increases in catalytic iron concentration in the spleen. Xiao et al. [33] also observed changes in nonheme iron and storage iron in the tissues of rats.

In our previous clinical study, we suggested that changes in the concentration of minerals under the influence of l-arginine may be linked to the impact of arginine in improving insulin secretion [6]. In that study, we observed decreases in copper serum concentration following l-arginine supplementation. The results of the present study show that the relation between copper and insulin following l-arginine treatment is rather indirect. However, the change in copper status is directly connected with the change in TAS and TNF alpha level. Other studies support the hypothesis of elevated iron stores, as well as inflammation and oxidative stress, in the development of insulin status disorders [6, 34, 35]. The changes in both iron and copper following the l-arginine supplementation observed in this study may be associated with metabolic relationships between these elements [36, 37]. Moreover, the concentration of iron and copper in serum and the tissues is associated with inflammatory and oxidative stress and with changes in the levels of these minerals involving amendments in the parameters of inflammation and antioxidant status [31, 38].

Our results here show the influence of l-arginine on insulin status and its effect on TAS and inflammation, which is strongly connected with the changes in the iron and copper status of rats fed with the high-fat diet. The direct mechanism responsible for the change in minerals following l-arginine supplementation needs further investigation.

The data obtained in this study is a new finding regarding the influence of l-arginine, with and without another antioxidant, on mineral metabolism in rats with a diet that promotes metabolic disorders.

This study has its limitations. We did not evaluate mineral levels in the serum of rats. Ferritin, ceruloplasmin, and mineral-dependent enzymes (e.g., superoxide dismutase and catalase), alongside other mineral metabolism parameters and inflammation and antioxidant markers, were not assessed. The determination of these parameters would allow a broader discussion on the impact of l-arginine on mineral status in rats fed with a high-fat diet. Moreover, in this experiment, the effect of combining l-arginine and vitamin C was studied on only one selected level. In further studies, it would be useful to use different doses of l-arginine in combination with other antioxidants.

In conclusion, the beneficial influence of l-arginine on insulin, TAS, and TNF alpha serum level is associated with changes in iron and copper status in rats fed a high-fat diet. The effect on mineral status and biochemical parameters of the administration of l-arginine with vitamin C is similar to that of supplementation with l-arginine alone in rats fed with the modified diet promoting negative metabolic changes.

## References

[1] Madero M, Perez-Pozo S, Jalal D, Johnson RJ, Sanchez-Lozada LG (2011). Dietary fructose and hypertension. Curr Hypertens Rep.

[2] Hao J, Liu SX, Zhao S, Liu QJ, Liu W, Duan HJ (2012). High-fat diet causes increased serum insulin and glucose which synergistically lead to renal tubular lipid deposition and extracellular matrix accumulation. Br J Nutr.

[3] Jacob PS, de Meneses Fujii TM, Yamada M, Borges MC, Pantaleao LC, Borelli P, Fock R, Rogero MM (2013). Isocaloric intake of a high-fat diet promotes insulin resistance and inflammation in Wistar rats. Cell Biochem Funct.

[4] Suliburska J (2013). A 6-week diet high in fat, fructose, and salt and its influence on lipid and mineral status, in rats. Acta Sci Pol Technol Aliment.

[5] De Luis DA, Pacheco D, Izaola O, Terroba MC, Cuellar L, Cabezas G (2011). Micronutrient status in morbidly obese women before bariatric surgery. Surg Obes Relat Dis.

[6] Suliburska J, Bogdanski P, Szulinska M, Pupek-Musialik D, Jablecka A (2013). Changes in mineral status are associated with improvements in insulin sensitivity in obese patients following l-arginine supplementation. Eur J Nutr.

[7] Wiernsperger N, Rapin J (2010). Trace elements in glucometabolic disorders: an update. Diabetol Metab Syndr.

[8] Rajapakse NW, Mattson DL (2009). Role of l-arginine in nitric oxide production in health and hypertension. Clin Exp Pharmacol Physiol.

[9] Piatti PM, Monti LD, Valsecchi G, Magni F, Setola E, Marchesi F, Galli-Kienle F, Pozza G, Alberti KG (2001). Long-term oral l-arginine administration improves peripheral and hepatic insulin sensitivity in type 2 diabetic patients. Diabetes Care.

[10] Loscalzo J (2004). l-arginine and atherothrombosis. J Nutr.

[11] Gokce N (2004). l-arginine and hypertension. J Nutr.

[12] Racasan S, Braam B, van der Giezen DM, Goldschmeding R, Boer P, Koomans HA, Joles JA (2004). Perinatal l-arginine and antioxidant supplements reduce adult blood pressure in spontaneously hypertensive rats. Hypertension.

[13] Bogdański P, Szulinska M, Suliburska J, Pupek-Musialik D, Jabłecka A, Witmanowski H (2013). Supplementation with l-arginine favorably influences plasminogen activator inhibitor type 1 concentration in obese patients. A randomized, double blind trial. J Endocrinol Investig.

[14] Bogdanski P, Suliburska J, Grabanska K, Musialik K, Cieslewicz A, Skoluda A, Jablecka A (2012). Effect of 3-month l-arginine supplementation on insulin resistance and tumor necrosis factor activity in patients with visceral obesity.. Eur Rev Med Pharmacol Sci.

[15] Reeves PG (1997). Components of the AIN-93 diets as improvements in the AIN-76A diet. J Nutr.

[16] Monti LD, Casiraghi MC, Setola E, Galluccio E, Pagani MA, Quaglia L, Bosi E, Piatti P (2013). l-arginine enriched biscuits improve endothelial function and glucose metabolism: a pilot study in healthy subjects and a cross-over study in subjects with impaired glucose tolerance and metabolic syndrome. Metabolism.

[17] Krause MS, McClenaghan NH, Flatt PR, de Bittencourt PI, Murphy C, Newsholme P (2011). l-arginine is essential for pancreatic β-cell functional integrity, metabolism and defense from inflammatory challenge. J Endocrinol.

[18] Thams P, Capito K (1999). l-arginine stimulation of glucose-induced insulin secretion through membrane depolarization and independent of nitric oxide. Eur J Endocrinol.

[19] Micker M, Chęciński P, Jabłecka A, Krauss H, Zapalski S (2002). Influence of l-arginine oral supplementation on oxidative stress in patients with intermittent claudication. Acta Angiol.

[20] Hosseini M, Pourganji M, Khodabandehloo F, Soukhtanloo M, Zabihi H (2012). Protective effect of l-arginine against oxidative damage as a possible mechanism of its beneficial properties on spatial learning in ovariectomized rats. Basic Clin Neurosci.

[21] Lucotti P, Monti L, Setola E, La Canna G, Castiglioni A, Rossodivita A, Pala MG, Formica F, Paolini G, Catapano AL, Bosi E, Alfieri O, Piatti P (2009). Oral l-arginine supplementation improves endothelial function and ameliorates insulin sensitivity and inflammation in cardiopathic nondiabetic patients after an aortocoronary bypass. Metabolism.

[22] Korish AA (2010). Multiple antioxidants and l-arginine modulate inflammation and dyslipidemia in chronic renal failure rats. Ren Fail.

[23] Mabalirajan U, Ahmad T, Leishangthem GD, Joseph DA, Dinda AK, Agrawal A, Ghosh B (2010). Beneficial effects of high dose of l-arginine on airway hyperresponsiveness and airway inflammation in a murine model of asthma. J Allergy Clin Immunol.

[24] El-Missiry MA, Othman AI, Amer MA (2004). l-arginine ameliorates oxidative stress in alloxan-induced experimental diabetes mellitus. J Appl Toxicol.

[25] Tousoulis D, Xenakis C, Tentolouris C, Davies G, Antoniades C, Crake T, Stefanidis C (2005). Effects of vitamin C on intracoronary l-arginine dependent coronary vasodilatation in patients with stable angina. Heart.

[26] Fan X, Xiaoqin L, Potts B, Strauch CM, Nemet I, Monnier VM (2011). Topical application of l-arginine blocks advanced glycation by ascorbic acid in the lens of hSVCT2 transgenic mice. Mol Vis.

[27] Fan X, Reneker LW, Obrenovich ME, Strauch C, Cheng R, Jarvis SM, Ortwerth BJ, Monnier VM (2006). Vitamin C mediates chemical aging of lens crystallins by the Maillard reaction in a humanized mouse model. Proc Natl Acad Sci U S A.

[28] Hegde KR, Varma SD (2004). Protective effect of ascorbate against oxidative stress in the mouse lens. Biochim Biophys Acta.

[29] Suliburska J (2013). The impact of iron content in a diet high in fat, fructose, and salt on metabolic state and mineral status of rats. J Physiol Biochem.

[30] Suliburska J, Bogdanski P, Szulinska M (2013). Iron excess disturbs metabolic status and relative gonad mass in rats on high fat, fructose, and salt diet. Biol Trace Elem Res.

[31] Steinbicker AU, Muckenthaler MU (2013). Out of balance-systemic iron homeostasis in iron-related disorders. Nutrients.

[32] Bo S, Durazzo M, Gambino R, Berutti C, Milanesio N, Caropreso A, Gentile L, Cassader M, Cavallo-Perin P, Pagano G (2008). Associations of dietary and serum copper with inflammation, oxidative stress, and metabolic variables in adults. J Nutr.

[33] Xiao DS, Jiang L, Che LL, Lu L (2003). Nitric oxide and iron metabolism in exercised rat with l-arginine supplementation. Mol Cell Biochem.

[34] Hassan WN, Mohamed MS, Ahmed E-SH, Abd-El-Mageed G, Morsy MD, Rouf AA (2010). Tumor necrosis factor-α and iron overload are associated with insulin resistance in hepatitis C in Egyptian patients. Am J Biomed Sci.

[35] Ashourpour M, Djalali M, Djazayery A, Eshraghian MR, Taghdir M, Saedisomeolia A (2010). Relationship between serum ferritin and inflammatory biomarkers with insulin resistance in a Persian population with type 2 diabetes and healthy people. Int J Food Sci Nutr.

[36] Syrovatka P, Kraml P, Potockova J, Fialova L, Vejrazka M, Crkovska J, Andel M (2009). Relationship between increased body iron stores, oxidative stress, and insulin resistance in healthy men. Ann Nutr Metab.

[37] Arredondo M, Nunez MT (2005). Iron and copper metabolism. Mol Asp Med.

[38] Rai KN, Kumari NS, Gowda KMD, Swathi KR (2013). Evaluation of micronutrients and oxidative stress and their relationship with the lipid profile in healthy adults. J Clin Diagn Res.

